# Genetic and Epigenetic Modification of Rat Liver Progenitor Cells via HNF4α Transduction and 5’ Azacytidine Treatment: An Integrated miRNA and mRNA Expression Profile Analysis

**DOI:** 10.3390/genes11050486

**Published:** 2020-04-29

**Authors:** Jennifer Bolleyn, Matthias Rombaut, Nisha Nair, Steven Branson, Anja Heymans, Marinee Chuah, Thierry VandenDriessche, Vera Rogiers, Joery De Kock, Tamara Vanhaecke

**Affiliations:** 1 Department of In Vitro Toxicology and Dermato-cosmetology (IVTD), Vrije Universiteit Brussel (VUB), Laarbeeklaan 103, B-1090 Brussels, Belgium; jennifer.bolleyn@hotmail.com (J.B.); Matthias.Rombaut@vub.be (M.R.); steven.branson@vub.be (S.B.); Anja.Heymans@vub.be (A.H.); Vera.Rogiers@vub.be (V.R.); Tamara.Vanhaecke@vub.be (T.V.); 2 Department of Gene Therapy and Regenerative Medicine (GTRM), Vrije Universiteit Brussel (VUB), Laarbeeklaan 103, B-1090 Brussels, Belgium; n7nair@gmail.com (N.N.); Marinee.Chuah@vub.be (M.C.); Thierry.Vandendriessche@vub.be (T.V.)

**Keywords:** liver-enriched transcription factor, azacytidine, microRNA, liver progenitor cell, lentiviral transduction, gene expression, rat liver epithelial cells

## Abstract

Neonatal liver-derived rat epithelial cells (rLEC) from biliary origin are liver progenitor cells that acquire a hepatocyte-like phenotype upon sequential exposure to hepatogenic growth factors and cytokines. Undifferentiated rLEC express several liver-enriched transcription factors, including the hepatocyte nuclear factors (HNF) 3β and HNF6, but not the hepatic master regulator HNF4α. In this study, we first investigated the impact of the ectopic expression of HNF4α in rLEC on both mRNA and microRNA (miR) level by means of microarray technology. We found that HNF4α transduction did not induce major changes to the rLEC phenotype. However, we next investigated the influence of DNA methyl transferase (DNMT) inhibition on the phenotype of undifferentiated naïve rLEC by exposure to 5′ azacytidine (AZA), which was found to have a significant impact on rLEC gene expression. The transduction of HNF4α or AZA treatment resulted both in significantly downregulated C/EBPα expression levels, while the exposure of the cells to AZA had a significant effect on the expression of HNF3β. Computationally, dysregulated miRNAs were linked to target mRNAs using the microRNA Target Filter function of Ingenuity Pathway Analysis. We found that differentially regulated miRNA–mRNA target associations predict ectopic HNF4α expression in naïve rLEC to interfere with cell viability and cellular maturation (miR-19b-3p/*NR4A2*, miR30C-5p/*P4HA2*, miR328-3p/*CD44*) while it predicts AZA exposure to modulate epithelial/hepatic cell proliferation, apoptosis, cell cycle progression and the differentiation of stem cells (miR-18a-5p/*ESR1*, miR-503-5p/*CCND1*). Finally, our computational analysis predicts that the combination of HNF4α transduction with subsequent AZA treatment might cause changes in hepatic cell proliferation and maturation (miR-18a-5p/*ESR1*, miR-503-5p/*CCND1*, miR-328-3p/*CD44*) as well as the apoptosis (miR-16-5p/BCL2, miR-17-5p/BCL2, miR-34a-5p/*BCL2* and miR-494-3p/*HMOX1*) of naïve rLEC.

## 1. Introduction

Historically, rat liver epithelial cells (rLEC) from biliary origin were used as helper cells in co-cultures of primary rat hepatocytes in order to restore their cell polarity and boost their functionality [1,2,3]. However, over the years, we have identified features of bipotent liver progenitor cells in naïve rLEC. More specifically, naïve rLEC stably express the progenitor markers CCAAT enhancer binding protein (C/EBP) α, hepatocyte nuclear factor (HNF) 3β, HNF6, connexin (Cx) 43, keratin (Krt) 18, Krt19 and stem cell factor receptor c-Kit [4,5]. Upon sequential exposure to hepatogenic growth factors and cytokines, rLEC generate functional hepatocyte-like cells, expressing (i) mature hepatic markers such as albumin (Alb), (ii) the active polarization of hepatic drug transporters such as the bile salt export pump (Bsep), the sodium/bile acid cotransporter (Ntcp), the multi-drug resistance protein 2 (Mrp2) and the sodium-independent organic anion-transporting polypeptide 4 (Oatp4), (iii) acquire the ability to store glycogen and (iv) display stable CYP1A1/2- and CYP2B1/2-dependent biotransformation capacity for several weeks at levels comparable to those observed in cultured primary rat hepatocytes [5]. These properties make rLEC a suitable tool to study the molecular mechanisms involved in the conversion of liver progenitor cells into hepatocytes.

In this study, we aimed to investigate the impact of genetic and epigenetic modifiers on the naïve rLEC phenotype by computationally identifying which molecular signaling pathways are altered using mRNA and miRNA expression profile analyses. First, we investigated the impact of ectopic HNF4α expression on the naïve rLEC phenotype as HNF4α plays a crucial role in early morphogenesis, fetal liver development, liver differentiation and metabolism and is not expressed in naïve rLEC [6,7]. As epigenetic events play a fundamental role in the determination of lineage-specific gene expression and cell fate, we subsequently exposed naïve rLEC to the DNA methyl transferase (DNMT) inhibitor 5′ azacytidine, alone or in combination with HNF4α transduction, in order to relieve epigenetically mediated gene silencing. These data will allow us to develop in the future more effective hepatic differentiation protocols for naïve rLEC.

## 2. Materials and Methods

### 2.1. Chemicals and Reagents

L-glutamine was purchased from Sigma-Aldrich (Overijse, Belgium). Williams’ E medium and FBS were obtained from Gibco BRL (Merelbeke, Belgium). All other chemicals and reagents were commercial products of the highest analytical grade available.

### 2.2. Animal Experiments

All procedures were performed in accordance with the ethical standards of the Vrije Universiteit Brussel and approved by its local Ethical Committee under grant number 9-210-1.

### 2.3. Vector Construction and Production

pLenti-GIII-CMV-GFP-2A-Puro plasmid was obtained from Applied Biological Material (Richmond, BC, Canada). The rat HNF4α cDNA was cloned downstream of the human cytomegalovirus virus (CMV) promoter. Lentiviral vectors were produced as previously described [8]. Briefly, 293T human embryonic kidney cells were cultured in Dulbecco’s modified Eagle medium supplemented with L-glutamine, pen/strep, and heat-inactivated fetal bovine serum (FBS). The cells were grown in single tray units (Nalgene Nunc International, Roskilde, Denmark). When the cells were 90% to 95% confluent, they were transfected by means of a calcium phosphate transfection protocol (Life Technologies, Merelbeke, Belgium). Plasmid DNA was extracted by means of Qiagen Maxi kits. The following DNA was used per single tray: transgene containing plasmid, pMDL gag/pol RRE helper plasmid, Rev-expressing plasmid, and pCI-VSV-G envelope-encoding plasmid. At 24 h after transfection, medium was removed and new medium with Nu-Serum IV instead of FBS (“serum-free” medium) supplemented with sodium butyrate (Sigma-Aldrich, Overijse, Belgium) was added per single tray. Conditioned medium was collected every 24 h during the subsequent 2 days. The lentiviral vectors containing conditioned media was concentrated by centrifugation at a speed of 2000 rpm at 4 °C for ~ 1 h using Centricon 70 plus filters. Lentiviral vector titration was calculated based upon a p24 assay (QuickTiter Lentivirus Quantitation Kit, Cellbiolabs, San Diego, CA, USA).

### 2.4. Isolation, Cultivation and Transduction of rLEC

rLEC were isolated from 8-day old male Sprague-Dawley rats, subcultivated and cryopreserved as previously described [9]. Briefly, small fragments of neonatal rat livers were incubated for 15 min with 4-(2-hydroxyethyl)-1-piperazine-ethanesulfonic acid (HEPES) buffered trypsin solution (0.25% (*v*/*v*)) and washed twice with calcium- and magnesium-free phosphate-buffered saline (PBS) before plating. The elimination of contaminating fibroblasts was accomplished by taking advantage of their faster attachment to plastic culture dishes (plate-and-wait method). Growth medium consisted of Williams’ E medium without glutamine, 10% (*v*/*v*) fetal bovine serum (FBS) (both from Life Technologies, Merelbeke, Belgium), 0.68 mM L-glutamine, 50 µg/mL streptomycin sulphate (both from Sigma-Aldrich, Overijse, Belgium), 7.33 IU/mL benzyl penicillin (Continental Pharma, Puurs, Belgium), 50 µg/mL kanamycin monosulphate (Sigma-Aldrich, Overijse, Belgium) and 10 µg/mL sodium ampicillin (Bristol–Meyers–Squibb, Brussels, Belgium). Cryopreserved rLEC, previously collected at passage (P) 12, were thawed and could be used up to P30 without loss of function as previously described [5]. Cell cultures were incubated at 37 °C in a 5% CO_2_ and 95% air humidified atmosphere. The growth media were changed completely every 2 days. All procedures were performed in accordance with the ethical standards of the Vrije Universiteit Brussel and approved by its local Ethical Committee. rLECs were transduced at 50% confluency with a viral titer of 2.09 × 10^9^ TU/mL. After transduction, the cells were subcultivated at 75% confluency. rLECs containing the lentiviral vectors encoding HNF4α and green fluorescent protein expression (GFP) or GFP alone (blank control vector) were puromycin selected. Puromycin (10 µg/mL; Sigma-Aldrich) was added to the growth medium and the cells were incubated at 37 °C for 24 h. After the puromycin selection, cells were washed and growth medium was changed. This was done every 2 days until 75% confluency was reached. This process of puromycin selection was performed twice. The experimental setup consisted of four culture conditions: (i) naïve rLEC (transduced with control vector), (ii) HNF4α-transduced rLEC, (iii) naïve rLEC exposed to a non-cytotoxic concentration of 20 µM 5′ azacytidine (Sigma-Aldrich, Overijse, Belgium) for 7 days (transduced with control vector) and (iv) HNF4α-transduced rLEC exposed to a non-cytotoxic concentration of 20 µM 5′ azacytidine for 7 days.

### 2.5. Hepatic Differentiation of rLEC

Naïve rLEC were cultivated at 100% confluency on 100 µg/mL rat tail collagen type I coated culture dishes (BD biosciences, Erembodegem, Belgium) in base medium and sequentially exposed to hepatogenic growth factors and cytokines. The base medium consisted of William’s E medium without glutamine (Life Technologies, Merelbeke, Belgium) supplemented with 7.33 IE/mL benzyl penicillin (Continental Pharma, Puurs, Belgium), 50 µg/mL streptomycin sulphate, 1 mg/mL linoleic-acid bovine serum albumin, 0.1 mM L-ascorbic acid, 0.03 mM nicotinamide, 0.25 mM sodium pyruvate and 1.623 mM L-glutamine (all from Sigma-Aldrich, Overijse, Belgium). The hepatic differentiation procedure was as follows: days 0–2: base medium + 2% (*v*/*v*) FBS (Life Technologies, Merelbeke, Belgium) + 20 ng/mL hepatocyte growth factor (HGF; R&D Systems, Abingdon, UK); days 3–5: base medium + 30 ng/mL HGF + 0.5% (*v*/*v*) insulin-transferrin-selenium (ITS; Sigma-Aldrich, Overijse, Belgium); day 6–8: base medium + 30 ng/mL HGF + 0.25% ITS + 20 µg/L dexamethasone (dex; Sigma-Aldrich, Overijse, Belgium); days 9–11: base medium + 20 ng/mL HGF + 20 µg/L dex; days 12–14: base medium + 10 ng/mL HGF + 20 µg/L dex + 10 ng/mL oncostatin M (OSM; R&D Systems, Abingdon, UK) and from day 15 onwards: base medium + 20 µg/L dex + 10 ng/mL OSM. Cell cultures were incubated at 33 °C in a 5% CO_2_ humidified atmosphere. Media were completely changed every three days, unless otherwise defined.

### 2.6. Isolation of Hepatocytes

Freshly isolated hepatocytes (for qPCR analysis) were isolated as previously described [10] by use of a two-step collagenase method from male outbred Sprague-Dawley rats (200–300 g), which were purchased from Charles River Laboratories (Brussels, Belgium). After verifying the viability (>80%) by trypan blue exclusion, the cells were snap frozen (−80 °C) until further analysis. 

### 2.7. Morphological analysis

Cell morphology was analyzed by light microscopy using a Nikon Eclipse Ti microscope (Nikon, Dilbeek, Belgium) with a 100× magnification. HNF4α transduction was examined by monitoring GFP.

### 2.8. Immunocytochemistry

Immunostainings were performed as previously described [11]. Briefly, cultivated cells were fixed with 4% (*w*/*v*) paraformaldehyde solution (PFA) for 10 min at room temperature and subsequently incubated for 15 min with 100 mM glycin solution, used to saturate reactive groups generated after PFA fixation (both from Sigma-Aldrich, Overijse, Belgium). Next, the cells were permeabilized for 15 min with 0.1% (*v*/*v*) Triton-X 100 solution (Sigma-Aldrich, Overijse, Belgium) in PBS and subsequently incubated for 45 min with blocking buffer consisting of 5% (*v*/*v*) serum of the respective host species of the secondary antibody (Jackson Immunoresearch, Cambridgeshire, UK). The cells were incubated with primary antibodies overnight at 4 °C. After four consecutive wash steps, the cells were incubated with fluorescence-labeled secondary antibodies for 90 min at room temperature. Finally, after washing thoroughly with 0.1% (*v*/*v*) Triton-X 100 solution in PBS (Sigma-Aldrich, Overijse, Belgium), the nuclei were stained with 4′,6-diamidino-2-phenylindole (DAPI) (Labconsult, Brussels, Belgium). The respective primary and secondary antibodies are listed in Table 1.

### 2.9. Flow Cytometry

Flow cytometric analyses were performed using an Attune Flow Cytometer (Life-Technologies, Merelbeke, Belgium). Briefly, 1 million naïve, GFP-transduced and GFP-HNF4α-transduced rLEC were harvested using TrypLE (Life-Technologies), subsequently washed with PBS and ran through the flow cytometer. Forward (FSC) and Side Scatter (SSC) parameters were used to select the rLEC population. Gating for GFP was done using GFP-negative (negative control) and GFP-positive (positive control, transduced with blank GFP vector) naïve rLEC. Approximately 100,000 events were collected to estimate the percentage of GFP-HNF4α-positive rLEC.

### 2.10. RNA Isolation

After culture, rLECs were harvested by scraping, washed twice with ice-cold PBS and pelleted thereafter. Total RNA enriched with small RNA (≤200 nt) was isolated using the mirVana™miRNA isolation kit (Ambion, Merelbeke, Belgium) according to the manufacturer’s instructions in an RNAse-free environment. The quantification of RNA in the samples was performed using a Nanodrop ND-1000 Spectrophotometer (NanoDrop Technologies, Wilmington, NC, USA).

### 2.11. Quantitative Real-Time PCR (qPCR)

Total RNA was reverse transcribed into cDNA using an iScript™ cDNA Synthesis Kit (BioRad, Temse, Belgium). The reverse transcriptase reaction consisted of 8 µL 5× iScript™ reaction mix, 2 µL iScript™ Reverse transcriptase and 30 µL RNA mixture, containing 2 µg RNA diluted in nuclease-free water. The reactions were incubated on a Biorad iCycler at 25 °C for 5 min, 42 °C for 30 min and 85 °C for 5 min. Following the RT step, cDNA purification was performed with the Genelute PCR clean up kit (Sigma–Aldrich, Overijse, Belgium) according to the manufacturer’s instructions. cDNA products were used for the quantitative amplification of the target genes. The primers used in this study are listed in Table 2. All samples were run in duplicate and each run included two no template controls (NTC) and a serial dilution of a pooled cDNA mix from all samples to calculate the efficiency curve. The qPCR reaction mix consisted of 12.5 μL TaqMan Universal Master Mix (Applied Biosystems, Merelbeke, Belgium), 1.25 μL 20× Assay-on-Demand Mix (Applied Biosystems, Merelbeke, Belgium) and 2 μL of cDNA in a 25 μL volume adjusted with nuclease-free water. qPCR conditions were as follows: enzyme activation for 2 min at 50 °C, incubation for 10 min at 95 °C, followed by 40 cycles of 15 s denaturation at 95 °C, annealing for 1 min at 60 °C, performed on a Bio-Rad iCycler iQ5 Multicolor Real-Time PCR (BioRad, Temse, Belgium). 

### 2.12. qPCR Data Analysis

For selecting reliable reference genes to normalize the qPCR data, we evaluated the expression stability of six candidate reference genes: glyceraldehyde 3-phosphate dehydrogenase (*GAPDH*), beta-2-microglobulin (*B2M*), hydroxymethylbilane synthase (*HMBS*), Eukaryotic 18S rRNA (*18S*), beta-actin (*ACTB*) and ubiquitin C (*UBC*). According to geNorm^®^, the optimal number of reference targets to be used in this experiment was 2 (V < 0.15). As such, *HMBS* and *ACTB* were selected as the most stable reference genes in all samples using qbasePLUS^®^ software (geNorm^®^, Biogazelle, Gent, Belgium). Relative mRNA expression levels were expressed as the fold changes normalized against the geometric means of both reference gene mRNAs using qbasePLUS^®^ software (Biogazelle, Zwijnaarde, Belgium). Statistical analyses were performed using a one-way unpaired ANOVA with Benjamini–Hochberg correction for multiple testing. Gene expressions with a fold change of at least two and a corrected p-value lower or equal to 0.05, were considered to be significantly different. 

### 2.13. Microarray Profiling of mRNAs

To evaluate the global mRNA expression, Affymetrix microarray technology was used. For each sample, 100 ng of total RNA was amplified and converted into biotinylated sense-strand DNA using the GeneChip^®^ WT PLUS Reagent Kit according to manufacturer’s instructions (Affymetrix, Merelbeke, Belgium). Next, samples were hybridized to a Rat transcriptome array 1.0 and placed in a GeneChip^®^ Hybridization Oven-645 (Affymetrix, Merelbeke, Belgium) rotating at 60 rpm at 45 °C for 16 h. After incubation, arrays were washed on a GeneChip^®^ Fluidics Station 450 and stained with the Affymetrix HWS kit in accordance with the manufacturer’s protocols. Finally, the arrays were scanned with an Affymetrix GeneChip^®^ Scanner 3000 7G (Affymetrix, Merelbeke, Belgium).

### 2.14. Microarray Profiling of MicroRNAs

The microarray profiling of miRNAs was performed using the same Affymetrix microarray technology. For each sample, 130 ng total RNA was labelled using the FlashTag^™^ Biotin HSR RNA Labeling Kit and subsequently hybridized to a GeneChip^®^ miRNA 4.0 Array. The arrays were subsequently placed at 48 °C in a GeneChip^®^ Hybridization Oven-645 rotating at 60 rpm for 16 to 18 h. After incubation, the arrays were washed and stained on a GeneChip^®^ Fluidics Station 450 using GeneChip^®^ Hybridization, Wash and Stain Kit according to the manufacturer’s instructions. The arrays were scanned with an Affymetrix GeneChip^®^ Scanner 3000 7G. 

### 2.15. Data Mining

Affymetrix^®^ Expression Console™ Software using Robust Multiarray Analysis (RMA) and detection above background (DABG) for data summarization, normalization and quality control was utilized. The data discussed in this publication have been deposited in NCBI’s Gene Expression Omnibus (GEO) and are accessible through GEO Series accession number GSE89250. For the determination of differential gene expression, output data files were analyzed using Affymetrix^®^ Transcriptome Analysis Console (TAC) software and Ingenuity Pathway Analysis (IPA, version 2019). Undifferentiated rLEC were compared to 5′ azacytidine (AZA)-treated and HNF4α-transduced rLEC (with and without AZA treatment) and evaluated for their expression of key hepatic mRNAs and miRNAs. mRNAs/miRNAs with a fold change > 2 and *p*-value < 0.05 were considered as significantly differentially expressed. Statistical analyses were performed using a one-way unpaired ANOVA with correction for multiple testing. The microRNA Target Filter function in IPA was used in order to link microRNA–mRNA interactions using experimentally validated interactions from TarBase and miRecords as well as microRNA-related findings from peer-reviewed literature.

## 3. Results

### 3.1. Ectopic HNF4α Expression Does Not Cause Major Changes to the Transcriptome of Naïve rLEC

**Figure 1:**
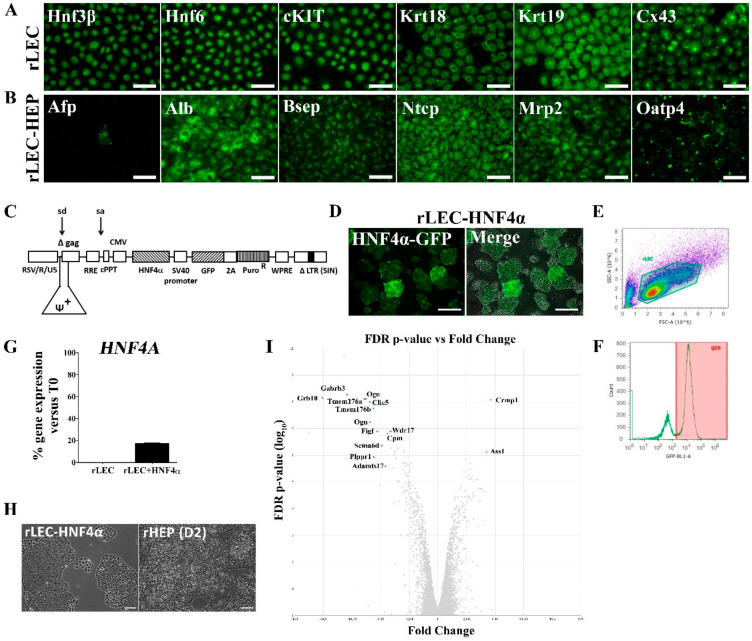
Transduction of naïve rLEC with HNF4α does not result in major transcriptome changes. (**A**) Naïve rLEC express liver progenitor markers and (**B**) acquire properties of hepatocyte-like cells upon sequential exposure to hepatogenic growth factors and cytokines. Scale bar: 50 µM. (**C**) A schematic representation of the lentiviral construct. (**D**) Transduced rLEC expressing GFP-HNF4α. The merge image includes corresponding phase contrast microscopy. Scale bar: 500 µM. E-F) Flow cytometric analysis of puromycin-purified rLEC after lentiviral transduction with GFP-HNF4α. (**G**) The endogenous expression of *HNF4A*. Freshly isolated rat hepatocytes (T0) are assigned the 100% expression for comparative and quantitative analysis. (**H**) The cell morphology of HNF4α-transduced naïve rLEC and primary rat hepatocytes (rHEP) 2 days in culture. Scale bar: 100 µM. (**I**) Volcano plot representing coding genes that are 5-fold upregulated (red) or down-regulated (green) in naïve rLEC upon ectopic HNF4α expression. Abbreviations: GFP: green fluorescent protein.

First, the hepatic progenitor status of our naïve rLEC cells was characterized as previously described [5]. We confirmed that our naïve rLEC stably expressed the progenitor markers HNF3β, HNF6, Cx43, Krt18, Krt19 and c-Kit (Figure 1A). Upon sequential exposure to hepatogenic growth factors and cytokines, naïve rLEC were able to generate functional hepatocyte-like cells, expressing Alb as well as the hepatic drug transporters Bsep, Ntcp, Mrp2 and Oatp4 (Figure 1B). Furthermore, in-depth, genome-wide comparative analyses of rLEC-derived hepatic cells with naïve rLEC revealed a significantly increased expression of several genes related to liver specific functional gene classes such as (a) carbohydrate metabolism, (b) drug metabolism, (c) lipid metabolism and (d) vitamin and mineral metabolism upon hepatic differentiation of naïve rLEC at comparable or higher levels than what is commonly found in freshly isolated rat hepatocytes (Figure S1). Ward’s hierarchical clustering showed a closer proximity between consecutive differentiation days for all four investigated functional gene classes. Importantly, a high correlation was observed between rLEC-derived hepatic cells from day 15 onwards (R = 0.996 ± 0.001; Figure S1). 

Next, to investigate the impact of the ectopic expression of the hepatic master regulator HNF4α, naïve rLEC were transduced with lentiviral vectors encoding GFP alone or GFP and HNF4α (Figure 1C; Figure S1). Widespread GFP expression was consistent with successful lentiviral transduction. Some variation in GFP expression was observed, possibly reflecting the variable integration of DNA copies (Figure 1D). Flow cytometric analysis showed that after puromycin exposure approximately 80% of the naïve rLEC cell line was GFP-positive (Figure 1E,F; Figure S2). Furthermore, we found that exogenous HNF4α transduction switched on endogenous HNF4α expression in the naïve rLEC, but not as high as in freshly isolated rat hepatocytes. More specifically, transduced rLEC only reached endogenous HNF4α expression levels of about 20% of that of adult rat hepatocytes (Figure 1G). The HNF4α-transduced rLEC maintained the epithelial cell shape of naïve rLEC, closely related to that of 2-day cultured primary rat hepatocytes (rHEP) (Figure 1H). Microarray analyses (24753 coding genes) revealed that 71 genes of the naïve rLEC transcriptome were significantly upregulated and 103 genes downregulated after lentiviral transduction with HNF4α, resulting in a modulation of gene expression of 0.70% of all coding genes. Using Ingenuity Pathway Analysis Software, we found that these modulated genes are involved in the canonical pathways ‘*Hepatic Fibrosis/Hepatic Stellate Cell Activation*’, ‘*PPARα/RXRα Activation*’, ‘*IGF-1 Signaling*’, ‘*VDR/RXR Activation*’, ‘*STAT3 Pathway*’, ‘*LXR/RXR Activation*’, ‘*PXR/RXR Activation*’, ‘*Glutathione-mediated Detoxification*’, ‘*Hepatic Cholestasis*’, ‘*BMP Signaling Pathway*’, ‘*Arginine Biosynthesis*’ and ‘*Hepatic Fibrosis Signaling Pathway*’ (Figure S2). Interestingly, the ‘*IGF-1 Signaling*’ pathway was predicted to be negatively influenced (z-score < −2; *p*-value < 0.05) by HNF4α over-expression (Figure S3).

Importantly, the expression of only two genes—collapsin response mediator protein 1 (*CRMP1*) and argininosuccinate synthase 1 (*ASS1*)—was more than 5-fold increased, whereas the expression of 12 genes—growth factor receptor bound protein 10 (*GRB10*), gamma-aminobutyric acid A receptor beta 3 (*GABRB3*), transmembrane proteins 176 A (*TMEM176A*) and B (*TMEM176B*), osteoglycin (*OGN*), phospholipid phosphatase related 1 (*PLPPR1*), c-fos induced growth factor (*FIGF*), sema domain 6 d (*SEMA6D*), ADAM metallopeptidase with thrombospondin type 1 motif 17 (*ADAMTS17*), carboxypeptidase M (*CPM*) and WD repeat domain 17 (*WDR17*)—was more than 5-fold decreased (Figure 1I).

### 3.2. AZA exposure Significantly Alters the Transcriptome of Naïve rLEC

**Figure 2:**
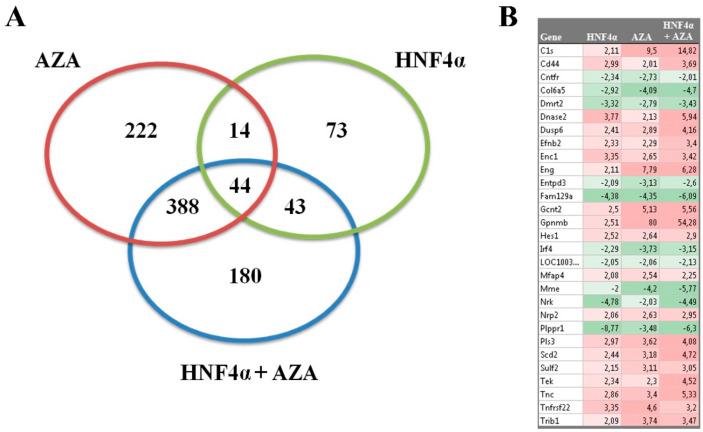
Significantly differentially expressed (fold change > 2, *p*-value < 0.05) coding genes in HNF4α-transduced, 5′ azacytidine (AZA)-treated and HNFα-transduced and AZA-treated rLEC compared to untreated control cells. (**A**) A Venn diagram showing common modulated genes between the different conditions. (**B**) A list of genes that are modulated in all three conditions in the same direction (up: red; down: green). Abbreviations: AZA: 5′ azacytidine; HNF: hepatocyte nuclear factor.

In this experiment, we exposed naïve and HNF4α-transduced rLEC to AZA for 7 consecutive days to ensure maximal effect of the demethylating agent [12]. Transcriptome analysis showed that 2.70% of the coding genes were modulated upon AZA treatment (215 genes downregulated, 453 genes upregulated). Combining HNF4α transduction with AZA exposure resulted in 200 downregulated and 455 upregulated coding genes, displaying the same magnitude in differential gene expression (2.65%) as AZA treatment alone. Interestingly, of the 655 differentially expressed genes in the combined condition, most of them, i.e., 432, were found to be linked to AZA treatment alone, whilst only 87 genes were linked to the HNF4α transduction condition (Figure 2A). Overall, 44 genes were modulated in all three culture conditions, of which 29 in the same direction (Figure 2B). 

A core analysis of the mRNA data could further identify different top molecular and cellular functions (Table 3) associated to the different experimental settings. As such, exposure of the rLECs to AZA resulted mainly in the differential expression of genes involved in *‘cellular growth and proliferation’* and *‘cell death and survival’*. Interestingly, the function *‘cellular growth and proliferation’* was also retrieved upon HNF4α transduction and the combined treatment. In addition, IPA analysis also predicts a significant effect of HNF4α transduction on the functional categories of *‘cellular assembly and organization’*, *‘gene expression’*, *‘cell-to-cell signaling and interaction’* together with *‘cellular compromise’*. The top molecular and cellular functions of the combined treatment mainly represent a reflection of the single AZA exposure culture condition.

Since the induction and maintenance of hepatic differentiation and the control of liver-specific gene expression are attributed to the coordinated expression of liver-enriched transcription factors (LETFs) [13], the outcome of HNF4α transduction and/or AZA exposure on the mRNA abundance of several LETF members was studied in more detail. As expected, and already shown by the qPCR data (Figure 1), microarray analysis confirms that endogenous *HNF4A* was significantly upregulated in HNF4α-transduced cultures (Table 4). Whilst AZA treatment alone did not significantly alter the *HNF4A* expression, additional exposure to the AZA of HNF4α-transduced rLEC further significantly augmented the *HNF4A* expression (Table 4). With respect to *HNF3B* (*FOXA2*), a significant increase in expression was only observed upon AZA treatment. As for CCAAT enhancer binding protein alpha *(CEBPA)*, both HNF4α transduction and AZA treatment significantly decreased its expression, but no synergistic effect was observed upon the combined treatment. No significant modulations were observed for *HNF1A* and *HNF6*.

### 3.3. MicroRNA Expression Profile of rLEC is Significantly Altered by HNF4α Transduction and AZA Exposure

**Figure 3:**
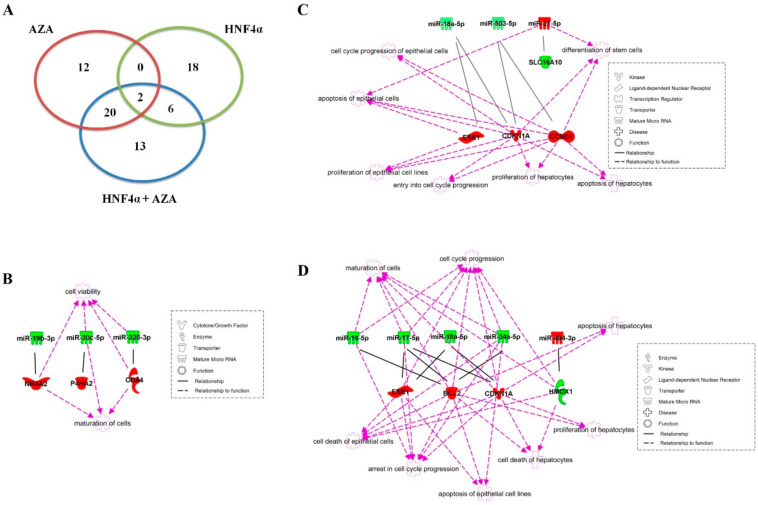
Computationally defined miRNA–mRNA interactions and their affected biological pathways in rLEC. (**A**) Significantly differential expressed (fold change >2, *p*-value <0.05) miRNAs in HNF4α-transduced, AZA-treated and HNF4α-transduced and AZA-treated rLEC compared to naïve rLEC. Subsequently, miRNA–mRNA interactions and their affected biological pathways were defined using Ingenuity Pathway Analysis miRNA Target Filter in naïve rLEC (**B**) transduced with HNF4α, (**C**) treated with 20µM 5′ azacytidine, and (**D**) transduced with HNF4α and subsequently exposed to AZA. The green color represents a downregulation, whereas red represents an upregulation. Abbreviations: AZA: 5′ azacytidine; BCL: B-cell lymphoma; CCND: Cyclin D; CD44: cluster of differentiation 44; CDKN: Cyclin-dependent kinase inhibitor; ESR: Estrogen receptor; HMOX: Heme oxygenase; HNF: hepatocyte nuclear factor; miR: microRNA; NR: Nuclear receptor; P4HA: Prolyl 4-hydroxylase; SLC: Solute carrier family.

When comparing the miRNA profiles of HNF4α-transduced rLEC with non-transduced rLEC, 26 miRNAs were found to be significantly differentially expressed (fold change > 2, *p*-value < 0.05). Interestingly, only three miRNAs were upregulated (Table S1). Exposure to 20 µM of the hypomethylating agent AZA, resulted in 34 differentially expressed miRNAs versus controls. Here, higher fold changes and an opposite trend, i.e., more upregulated miRNAs as compared to HNF4α-transduced cells were observed. Upon the combined action of HNF4α transduction and AZA exposure, 41 miRNAs were significantly differentially expressed. In total, 20 and six of these miRNAs were found to be common to AZA treatment and HNF4α transduction alone, respectively (Figure 3A). Interestingly, only two miRNAs, i.e., miR-34a-5p and miR-6215, could be identified as common differentially expressed in HNF4α-transduced cells, AZA-treated and the combination of both. In case of miR-6215, a downregulation was observed. However, for miR-34a-5p, an upregulation was observed in AZA-treated cells, whilst the opposite effect, i.e., downregulation, was noticed in HNF4α-transduced cells. The latter downregulation remained upon additional AZA exposure. 

Using the microRNA Target Filter function of IPA, possible miRNA–mRNA target connections were identified and computationally linked to pathways associated with hepatic cells, epithelial cells and stem cells. As such, for HNF4α-transduced rLEC, three miRNAs, i.e., miR-19b-3p, miR-30c-5p and miR-382-3p, were shown to be inversely correlated with nuclear receptor *NR4A2*, prolyl 4-hydroxylase 2 (*P4AH2*) and the cluster of differentiation 44 (*CD44*), respectively (Figure 3B). Those miRNA–mRNA networks are predicted to affect cell maturation and viability of rLEC. Upon AZA treatment of naïve rLEC, three miRNAs (miR-18a-5p, miR-503-5p and miR-21-5p) were linked to four mRNA targets using the same *in silico* approach—estrogen receptor 1 (*ESR1*), cyclin-dependent kinase inhibitor 1A (*CDKN1A*), cyclin D1 (*CCND1*) and solute carrier family *SLC16A10* (Figure 3C). These mRNA targets are known to play a role in several biological pathways, including epithelial/hepatic proliferation, apoptosis and cell cycle progression and the differentiation of stem cells. Finally, combining HNF4α transduction with AZA treatment resulted in five miRNAs (miR-16-5p, miR-17-5p, miR-18a-5p, miR-34a-5p and miR-494-3p) that were computationally linked to four mRNA targets (*ESR1*, b-cell lymphoma 2 (*BCL2*), *CDKN1A* and heme oxygenase 1 (*HMOX1*) (Figure 3D). Two of those mRNAs (*ESR1* and *CDNK1A*) and one miRNA species (miR-18a-5p) were also identified upon single AZA treatment. The combined treatment of HNF4α and AZA resulted in identified miRNA–mRNA interactions that were predicted to affect the maturation of cells and processes involved in cell cycle arrest. Cell cycle progression, cell death and proliferation were also identified. In Table 5, the fold change expression ratio of all the identified target mRNAs versus controls, as observed in the mRNA microarray analysis, are shown. Interestingly, the expression of *NR4A2*, *P4AH2* and *CD44* was found to be significantly changed in all culture conditions and an average fold change expression of the ratios found for single AZA-treated and HNF4α-transduced cells was seen in the combination of both treatments for *P4HA2*. For *NR4A2* and *CD44*, the combination of both treatments resulted in a synergistic effect on the fold change expression.

## 4. Discussion

rLEC were previously used as helper cells in co-cultures to support long-term hepatic functionality [1,2]. In addition, these cells share similar properties with bile ductular epithelial cells and oval cells and are able to differentiate towards hepatocyte-like cells [5,14]. As demonstrated by De Kock et al., these cells possess several markers of bipotent liver progenitor cells, including the LETFs C/EBPα, HNF3β and HNF6 [5]. Using a sequential exposure protocol to hepatogenic growth factors and cytokines, the same authors could also show that the cells displayed a hepatocyte-like phenotype, including biotransformation capacity [5]. 

In this study, we first aimed at assessing changes in the gene expression profile of naïve rLEC upon the ectopic expression of HNF4α. This transcription factor, which is of utmost importance to maintain and promote the hepatic phenotype in hepatocytes [6], is not expressed by naïve rLEC [5]. Together with other LETFs, HNF4α plays an elemental role in hepatocyte-specific gene expression and is as such a key regulator in liver development, architecture and physiology [15]. It has already been shown that HNF4α is essential for the specification of hepatic progenitors derived from human pluripotent stem cells [16]. The concept of engineering cell fate and phenotype changes by the overexpression of key transcription factors was already reported to be successful. Indeed, Iacob and colleagues could show that a sequential ectopic expression of HNF3β (also known as FoxA2), HNF4α and C/EBPα induced a hepatic phenotype in an expendable liver-derived progenitor cell line from mouse origin [13]. 

Since differentiation processes are largely accompanied by epigenetic modification of genetic regions, we also exposed the cells to 20 µM 5′ azacytidine in order to study the effects on the gene expression profile of both naïve and HNF4α-transduced rLEC [15,17,18]. Moreover, it has been shown that the use of DNMTi could trigger cell cycle arrest and promote cellular differentiation in HepG2 cells [15,19]. Furthermore, the addition of DNMTi was shown to significantly improve hepatic features, including phase I and II enzyme activities and higher expression of transcription factors in different cell lines, including HeLa cells, human hepatoma cells and mouse hepatocytes [15,18]. Using human mesenchymal stem cells, Tsai and colleagues reported that another DNMTi, called 5-aza-2′-deoxycitidine, together with the histone deacetylase inhibitor (HDACi) trichostatin A (TSA), protects the cells from cell death during their differentiation towards hepatocytes and triggers a wide range of liver-specific markers as well as liver functions [19]. Similar results could, however, not be found when applying the same epigenetic regulators to rat bone marrow-derived mesenchymal stem cells [20]. This indicated that epigenetic gene regulation is cell type specific. Upon hepatic differentiation, naïve rLEC undergo gene expression changes in lipid, carbohydrate, vitamin and mineral, and drug metabolism, as shown by our transcriptomics data. Interestingly, we also found that AZA treatment modulated the expression of naïve rLEC genes involved in ‘*Lipid metabolism*’ and ‘*Small molecule biochemistry*’. Therefore, AZA treatment might be considered to further improve the hepatic differentiation of rLEC in subsequent optimization attempts of the hepatic differentiation protocol.

### 4.1. Genome-Wide Analysis of Both miRNA and mRNA Expression

In this study, we performed a genome-wide analysis of both miRNA and mRNA expression in order to explore the effects of HNF4α transduction and/or AZA exposure on the rLEC transcriptome. Comparative analysis between AZA cultures and control cultures resulted in the identification of 34 differentially expressed miRNA species and 668 differentially expressed annotated coding genes. HNF4α transduction, on the other hand, resulted in the differential expression of 26 miRNAs and 174 genes, pointing towards a greater impact of epigenetic regulation than ectopic expression on both miRNA and mRNA expression profiles. The same impact on mRNA levels, as witnessed in the AZA condition, was also retrieved in the HNF4α and AZA condition. Remarkably, only two miRNA species were found to be differentially regulated in AZA-treated (upregulation) versus HNF4α-transduced cells (downregulation), i.e., rno-miR-34a-5p and rno-miR-6215. Others could already show that miR-34a is a negative regulator of HNF4α in HepG2 cells, [21,22,23]. Recently, Park and colleagues found that this miRNA species is involved in the differentiation of human adipose tissue-derived stem cells. Indeed, by overexpressing miR-34a, cell proliferation was inhibited due to the downregulation of several cell cycle regulators, such as cyclin-dependent kinases 2, 4 and 6, and cyclins E and D [24]. In this study, computational analyses also predicted miR-34a as a regulator of CDKN1, an important regulator in cell death and cell cycle progression in HNF4α-transduced + AZA-treated cells. 

### 4.2. Evaluation of the LETFs Network

Next, focus was set on the gene expression levels of several LETFs. The combinatorial actions of these transcription factors lead to the dynamic regulation of gene expression required for a proper differentiation of hepatocytes [13,25]. Although HNF4α was successfully transduced into rLEC, only approximately 20% of the endogenous mRNA levels could be obtained when compared to freshly isolated rat hepatocytes. HNF4α transduction as well as AZA treatment resulted in a significant, yet modest, downregulation of *CEBPA*, but no synergistic effect was observed upon the combined treatment. C/EBPα is known to be responsible for maintaining the differentiated status of hepatocytes and directs transcription of many liver-specific genes. The observed modest downregulation of *CEBPA* might interfere with the hepatic maturation capacity of naïve rLEC when treated with AZA or transduced with HNF4α. Importantly, although only modest changes to the rLEC transcriptome were observed upon HNF4α transduction, *ASS1*, the argininosuccinate synthetase enzyme that plays a prominent role in the urea cycle of hepatocytes, was found to be more than 5-fold upregulated [26]. AZA treatment on the other hand resulted in an upregulation of *HNF3B*. HNF3β was previously shown to act in a cooperative, synergistic regulatory network with HNF6 that affects hepatocyte-specific gene transcription [15,25,27]. However, we did not observe an effect on *HNF6* and *HNF1A* expression. HNF3β, also known as FoxA2, is also a pioneering factor, like FoxA3, required for successful direct reprograming of fibroblasts into so-called induced hepatocytes (iHeps) [28]. An increased expression of *HNF3B* might therefore be beneficial for the hepatic differentiation and maturation of naïve rLEC.

### 4.3. Linking miRNA–mRNA Target Interactions to Functional Pathways

Computational analyses of differentially expressed miRNA–mRNA target associations predicted that the treatment of naïve rLEC with AZA might modulate cell proliferation supported by the downregulation of miR-18a-5p that could be linked to an upregulation of both *CDKN1A* and *ESR1*. Moreover, the downregulation of miR-18a has previously been shown to induce growth retardation in neuroblastoma cells via inhibition of *ESR1* mRNA expression [29]. The same inversely correlated relationship was also observed in hepatocellular carcinoma (HCC) cells where the overexpression of miR-18a caused a downregulation of *ESR1* and a stimulation of proliferation [30]. Next, our computational analyses also predicted that upregulated miR-503-5p might repress *CCND1* mRNA expression. In breast cancer cells, the overexpression of this miR-503 was shown to reduce cell proliferation through the induction of a G1 cell cycle arrest by targeting *CCND1* [31]. The same interaction was also witnessed in endometrial cancer cells [32]. Interestingly, in our study, *‘cellular growth and proliferation’* together with *‘cell death and survival’* were identified as top molecular and cellular functions in the IPA core analysis, respectively. 

Upon HNF4α transduction, 3 miRNA–mRNA target pairs were predicted by *in silico* analysis, one of them being miR-328-3p that inversely correlated with *CD44*. In renal tubular cells, miR-328 was found to play a role in the epithelial-to-mesenchymal transition (EMT) by targeting *CD44* [33]. EMT together with the reverse transdifferentiation event, also known as mesenchymal-to-epithelial transition (MET) has been proposed as key elements of stem cell biology [34,35]. Indeed, the presence of both epithelial and mesenchymal traits defines the precursor phenotype. In hepatocytes, EMT has been related to the dedifferentiation process in freshly isolated hepatocytes, together with the downregulation of the hepatic differentiation key factor HNF4α. HNF4α was identified to be the dominant regulator in the epithelial phenotype [35]. Overexpression of HNF4α was also found to be sufficient in the re-establishment of the hepatocyte marker gene expression, epithelial cell morphology and polarity in dedifferentiated hepatoma cells [36]. 

The combination of HNF4α transduction and AZA exposure identified 5 important miRNAs targeting 4 different mRNAs. At first, three miRNAs (miR-16-5p, miR-17-5p and miR-34a-5p) were predicted to target *BCL2*, being an important regulator of cell death [37]. In an acute liver failure setting, it was shown that miR-16 reduced hepatic apoptosis via the anti-apoptotic gene *BCL2* [38]. *BCL2* was also identified as a target of the miR-17-92 family in leukemia [39]. As for miR-34a, its overexpression in pancreatic β cells was found to induce apoptosis [40]. Also, in non-small cell lung cancers, miR-34a was identified to play a role in apoptosis by targeting *BCL2* [41]. miR-17-5p and miR-18a-5p were predicted to target both *ESR1* and *CDKN1A*. This link between miR-18a-5p and the *ESR1* and *CDKN1A* mRNAs was also predicted in naïve rLEC cultures exposed to AZA and is suggested to have an impact on cell proliferation. miR-494-3p was the only upregulated miRNA that was inversely correlated with *HMOX1*, known to play a role in apoptosis [42]. A correlation of this miRNA–mRNA pair was also found previously in ischemic brain [43]. 

## 5. Conclusions

This study represented a computational and integrated analysis of miRNA and mRNA expression profiles in HNF4α-transduced and/or AZA-treated naïve rLEC. We identified a number of miRNAs and transcripts that were differentially expressed upon the respective treatments. In case of AZA treatment, the differential expression of several miRNAs that are inversely correlated with factors that are involved in epithelial/hepatic cell proliferation (miR-18a-5p/*ESR1*, miR-503-5p/*CCND1*), was observed. As for HNF4α transduction, identified miRNA–mRNA pairs (miR-328-3p/*CD44*) were predicted to be involved in cell maturation and viability. HNF4α transduction together with AZA treatment introduced changes in the rLEC transcriptome that were predicted to be involved in hepatic cell proliferation (miR-18a-5p/*ESR1*, miR-503-5p/*CCND1*), apoptosis (miR-16-5p/*BCL2*, miR-17-5p/*BCL2*, miR-34a-5p/*BCL2* and miR-494-3p/*HMOX1*) and cellular maturation (miR-328-3p/*CD44*). Altogether, this study provides new data on the alteration of the naïve rLEC transcriptome using genetic and epigenetic modifiers and is of importance for future optimization of the *in vitro* differentiation and maturation of rLEC into fully functional hepatocyte-like cells. 

## Figures and Tables

**Table 1:** Primary and secondary antibodies used for the characterization of naïve rat liver epithelial cells (rLEC) and rLEC-derived hepatic cells.

Antibody	Type	Order ID	Species	Dilution	Source
anti-Afp	polyclonal	sc-8108	goat	1/50	SC
anti-Hnf3β	polyclonal	sc-6554	goat	1/50	SC
anti-c-kit	polyclonal	sc-1494	goat	1/50	SC
anti-Oatp4	polyclonal	sc-134461	rabbit	1/50	SC
anti-Ntcp	polyclonal	sc-107029	goat	1/50	SC
anti-Hnf6	polyclonal	sc-13050	rabbit	1/50	SC
anti-Cx43	polyclonal	C6219	rabbit	1/100	SA
anti-Krt18	monoclonal	F4772	mouse	1/50	SA
anti-Krt19	monoclonal	C7159	mouse	1/20	SA
anti-Mrp2	monoclonal	sc-59611	mouse	1/50	SC
anti-Bsep	monoclonal	sc-74500	mouse	1/50	SC
anti-Alb	polyclonal	A110-125	rabbit	1/50	BL
anti-rabbit	Dylight-488	711-485-152	donkey	1/500	JI
anti-mouse	Dylight-488	715-485-150	donkey	1/500	JI
anti-goat	Dylight-488	711-485-152	donkey	1/500	JI

Abbreviations: alpha-fetoprotein (Afp); albumin (Alb); Bethyl Laboratories (BL); Bile salt export pump (Bsep); connexin (Cx); hepatocyte nuclear factor (Hnf); Jackson Immunoresearch (JI); keratin (Krt); Multi-drug resistance protein (Mrp); Na+/bile acid cotransporter (Ntcp); organic anion-transporting polypeptide (Oatp); stem cell growth factor receptor (c-kit); Santa Cruz (SC); Sigma-Aldrich (SA).

**Table 2:** Primers used for the evaluation of the gene expression of housekeeping genes and gene encoding for endogenous HNF4α.

Gene	Assay-On-Demand ID	Amplicon Length (bp)
*GAPDH*	Rn01775763_g1	174
*B2M*	Rn00560865_m1	58
*UBC*	Rn01789812_g1	88
*ACTB* *	Rn00667869_m1	91
*18S*	Hs99999901_s1	187
*HMBS* *	Rn00565886_m1	99
*HNF4A*	Rn04339144_m1	53

Abbreviations: 18S: Eukaryotic 18S rRNA; ACTB: beta-actin; B2M: beta-2-microglobulin; GAPDH: glyceraldehyde 3-phosphate dehydrogenase; HMBS: hydroxymethylbilane synthase; HNF: hepatocyte nuclear factor; UBC: ubiquitin C. * selected reference genes.

**Table 3:** Top affected molecular and cellular functions together with the number of differentially expressed genes in the respective pathways.

AZA		HNF4α Transduction		HNF4α Transduction + AZA	
Molecular and Cellular Functions	Amount of Genes	Molecular and Cellular Functions	Amount of Genes	Molecular and Cellular Functions	Amount of Genes
Cellular growth and proliferation	91	Cellular assembly and organisation	15	Cellular growth and proliferation	95
Cell death and survival	76	Cellular compromise	8	Cell death and survival	73
Cellular movement	37	Cellular growth and proliferation	20	Cellular movement	37
Lipid metabolism	29	Gene expression	13	Cellular assembly and organisation	57
Small molecule biochemistry	17	Cell-to-cell signaling and interaction	16	Cellular function and maintenance	59

Abbreviations: AZA: 5′ azacytidine; HNF: hepatocyte nuclear factor.

**Table 4:** mRNA expression of endogenous liver-enriched transcription factors in HNF4α-transduced, AZA-treated and HNF4α-transduced and AZA-treated rat liver progenitor cells compared to untreated control cells.

	HNF4α Transduction	AZA	HNF4α Transduction + AZA
Gene	Fold Change	Fold Change	Fold Change
***HNF1A***	1.01	1.01	−1.01
***HNF3B (FOXA2)***	1.1	1.83 *	1.54*
***HNF4A***	2.88 *	−1.03	4.37 *^$^
***HNF6***	1.04	1.09	−1.04
***CEBPA***	−1.38 *	−1.37 *	−1.36 *

* Fold change was significantly different between treated and control cultures (*p*-value < 0.05). $ Fold change was significantly different between HNF4α-transduced and HNF4α-transduced + AZA-treated cells (*p*-value < 0.05). Abbreviations: AZA: 5′ azacytidine; CEBPA: CCAAT enhancer binding protein alpha; HNF: hepatocyte nuclear factor.

**Table 5:** Fold change expression ratio of all the identified target mRNAs in HNF4α-transduced, AZA-treated and HNF4α-transduced and AZA-treated rat liver progenitor cells compared to untreated control cells.

	HNF4α Transduction		AZA		HNF4α Transduction + AZA	
Gene	Fold Change	*p*-Value	Fold Change	*p*-Value	Fold Change	*p*-Value
*BCL2*	1.36 *	0.001289	1.78 *	0.000086	**2.47** *	0.000012
*CCND1*	1.08	0.806816	**2.26** *	0.000349	1.77 *	0.000081
*CD44*	**2.99** *	0.000012	**2.01** *	0.000148	**3.69** *	0.000019
*CDKN1A*	1.76 *	0.018413	**17.19** *	0.000020	**3.4** *	0.000802
*ERS1*	1.01	0.834552	**2.35** *	0.000142	**2.72** *	0.000082
*HMOX1*	−1.02	0.954842	−**2.98** *	0.004230	−**2.99** *	0.001001
*NR4A2*	**3.77** *	0.019828	**5.16** *	0.009264	**9.17** *	0.002329
*P4HA2*	**2.19** *	0.000041	−**2.27** *	0.000048	1.14 *	0.001325
*SLC16A10*	1.19	0.261210	−**2.58** *	0.001054	−1.23 *	0.011956

* Fold change was significantly different between treatment and control cultures (*p*-value < 0.05). Fold changes in bold were found to be larger than the cut-off value of 2. Abbreviations: AZA: 5′ azacytidine; BCL: B-cell lymphoma; CCND: CCND: Cyclin D; CD: cluster of differentiation; CDKN: cyclin-dependent kinase inhibitor; ESR: Estrogen receptor; HMOX: Heme oxygenase; HNF: hepatocyte nuclear factor; NR: nuclear receptor; P4HA: prolyl 4-hydroxylase; SLC: solute carrier family.

## Supplementary Materials

The following are available online at https://www.mdpi.com/2073-4425/11/5/486/s1, Figure S1: In-depth genome wide comparative analysis of rLEC-derived hepatic cells with naïve rLEC; Figure S2: Transduction of naïve rLEC with blank GFP control lentiviral vector; Figure S3: Canonical pathways modulated in HNF4α-transduced rLEC; Table S1: Significantly differentially expressed miRNAs in HNF4α-transduced, AZA-treated and HNF4α-transduced + AZA-treated rat liver progenitor cells compared to untreated control cells.
