# Supplementary material for: Genetic and Epigenetic Modification of Rat Liver Progenitor Cells via HNF4α Transduction and 5’ Azacytidine Treatment: An Integrated miRNA and mRNA Expression Profile Analysis
Source: Genes (Basel). 2020 Apr 29;11(5):486. doi: 10.3390/genes11050486 (PMC7291069; doi:10.3390/genes11050486)
Supplement: Supplementary file 1 [file genes-11-00486-s001.pdf]

Genetic and epigenetic modification of rat liver progenitor cells *via* HNF4α transduction and 5' azacytidine treatment: an integrated miRNA and mRNA expression profile analysis

Jennifer Bolleyn, Matthias Rombaut, Nisha Nair, Steven Branson, Anja Heymans, Marinee Chuah, Thierry VandenDriessche, Vera Rogiers, Joery De Kock and Tamara Vanhaecke

Table S1: Significantly differentially expressed (fold change >2, p-value <0.05) miRNAs in HNF4α-transduced, AZA-treated and HNF4α-transduced + AZA-treated rat liver progenitor cells compared to untreated control cells.

AZA			HNF4α transduction			HNF4α transduction + AZA		
**Transcript ID**	**Fold Change**	**p-value**	**Transcript ID**	**Fold Change**	**p-value**	**Transcript ID**	**Fold Change**	**p-value**
rno-miR-741-3p	43.65	0.000007	rno-miR-335	4.70	0.007744	rno-miR-741-3p	48.19	0.000003
rno-miR-743a-3p	23.70	0.000147	rno-miR-3473	2.90	0.002781	rno-miR-743a-3p	26.09	0.000050
rno-miR-871-3p	14.42	0.000015	rno-miR-509-5p	2.06	0.041749	rno-miR-21-5p	23.28	0.011940
rno-miR-878	14.29	0.000043	rno-miR-328a-3p	-2.03	0.035641	rno-miR-871-3p	16.03	0.000206
rno-miR-21-5p	10.91	0.020745	rno-miR-342-5p	-2.03	0.023492	rno-miR-878	11.05	0.000073
rno-miR-130b-3p	6.71	0.000580	rno-miR-20b-5p	-2.04	0.044670	rno-miR-3580-3p	7.60	0.000138
rno-miR-23b-5p	6.39	0.005097	rno-miR-19b-3p	-2.07	0.008338	rno-miR-199a-3p	6.06	0.001292
rno-miR-3580-3p	6.28	0.002151	rno-miR-126a-3p	-2.07	0.000690	rno-miR-429	5.38	0.004102
rno-miR-34a-5p	5,99	0.009567	rno-miR-195-5p	-2,11	0.000539	rno-miR-130b-3p	5,28	0.000363
rno-miR-675-3p	5,84	0.000819	rno-miR-483-3p	-2,19	0.033207	rno-miR-27a-5p	4,72	0.002141
rno-miR-871-5p	5.69	0.033350	rno-miR-345-3p	-2.25	0.005064	rno-miR-30e-3p	4.58	0.010396
rno-miR-429	4.78	0.000493	rno-miR-138-1-3p	-2.26	0.010835	rno-miR-146a-5p	4.13	0.000494
rno-miR-27a-5p	4.64	0.004763	rno-miR-22-3p	-2.38	0.012907	rno-miR-871-5p	3.81	0.002212

rno-miR-3593-3p	4.63	0.015812	rno-miR-298-5p	-2.39	0.029185	rno-miR-132-3p	3.70	0.000603
rno-miR-378a-5p	3.65	0.049934	rno-miR-3596a	-2.53	0.000400	rno-miR-675-3p	3.70	0.014021
rno-miR-675-5p	3.17	0.028217	rno-miR-6215	-2.67	0.000865	rno-miR-463-3p	3.20	0.000780
rno-miR-742-3p	3.03	0.003612	rno-miR-30b-5p	-3.32	0.005837	rno-miR-675-5p	3.20	0.027242
rno-miR-30e-3p	3.01	0.003000	rno-miR-140-5p	-3.34	0.044668	rno-miR-200b-3p	3.08	0.003434
rno-miR-463-3p	2.89	0.000980	rno-miR-195-3p	-3.49	0.028040	rno-miR-664-2-5p	2.92	0.006014
rno-miR-27b-5p	2.83	0.032424	rno-miR-196b-5p	-3.59	0.006382	rno-miR-326-5p	2.86	0.029265
rno-miR-143-3p	2.73	0.005212	rno-miR-34a-5p	-3.69	0.032786	rno-miR-212-3p	2.84	0.016886
rno-miR-212-3p	2.67	0.020855	rno-miR-296-3p	-3.78	0.011440	rno-miR-6318	2.79	0.026361
rno-miR-200c-3p	2.59	0.024949	rno-miR-872-3p	-3.86	0.022192	rno-miR-27b-5p	2.39	0.033939
rno-miR-200b-3p	2.58	0.008496	rno-miR-30e-5p	-5,00	0.005305	rno-miR-203a-3p	2.37	0.031911
rno-miR-346	2.32	0.046358	rno-miR-301a-3p	-8.87	0.005423	rno-miR-92b-5p	2.33	0.035394
rno-miR-132-3p	2.20	0.004007	rno-miR-497-5p	-10.62	0.001091	rno-miR-494-3p	2.20	0.015707
rno-miR-146a-5p	2.02	0.025522				rno-miR-3473	2.17	0.008224
rno-miR-23b-3p	2.01	0.000505				rno-miR-194-5p	2.15	0.039591
rno-miR-27b-3p	2.01	0.000576				rno-miR-200b-5p	2.02	0.040727
rno-miR-183-3p	-2.13	0.039336				rno-miR-1839-5p	2.02	0.030531
rno-miR-503-5p	-2.36	0.000542				rno-miR-195-5p	-2.10	0.000010
rno-miR-18a-5p	-2.38	0.001716				rno-miR-20b-5p	-2.19	0.026821
rno-let-7d-3p	-2.64	0.015201				rno-miR-20a-5p	-2.23	0.000033
rno-miR-6215	-4.32	0.011050				rno-miR-3102	-2.36	0.016359
						rno-miR-18a-5p	-2.57	0.001904
						rno-miR-6215	-3.11	0.003128
						rno-miR-188-5p	-3.12	0.018297
						rno-miR-34a-5p	-3.16	0.048781
						rno-miR-195-3p	-3.58	0.046002
						rno-miR-301a-3p	-3.77	0.013753
						rno-miR-497-5p	-6.15	0.000333

Abbreviations: AZA: 5' azacytidine; HNF: hepatocyte nuclear factor; miR: microRNA; rno: rattus norvegicus.

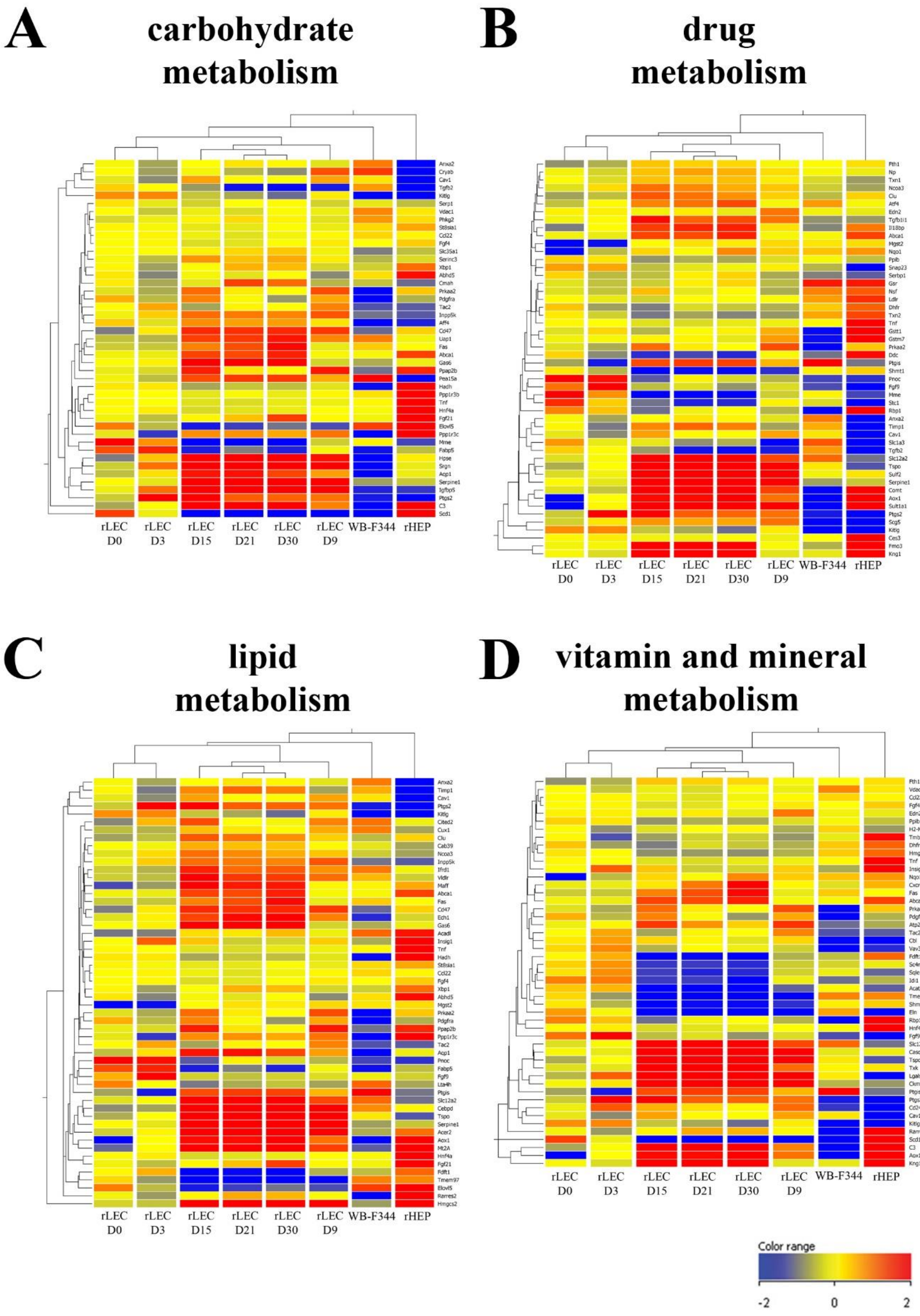

Figure S1 In-depth genome wide comparative analysis of rLEC-derived hepatic cells with naïve rLEC. Transcriptome analysis reveals a significantly increased expression of several genes related to liver specific functional gene classes such as A) carbohydrate metabolism, B) drug metabolism, C) lipid metabolism and D) vitamin and mineral metabolism upon hepatic differentiation of naïve rLEC at comparable or higher levels than what is commonly found in freshly isolated rat hepatocytes. Ward's

hierarchical clustering shows a closer proximity between consecutive differentiation days for all 4 investigated functional gene classes. Importantly, a high correlation is observed between rLEC-derived hepatic cells from day 15 onwards (R = 0.996±0.001).

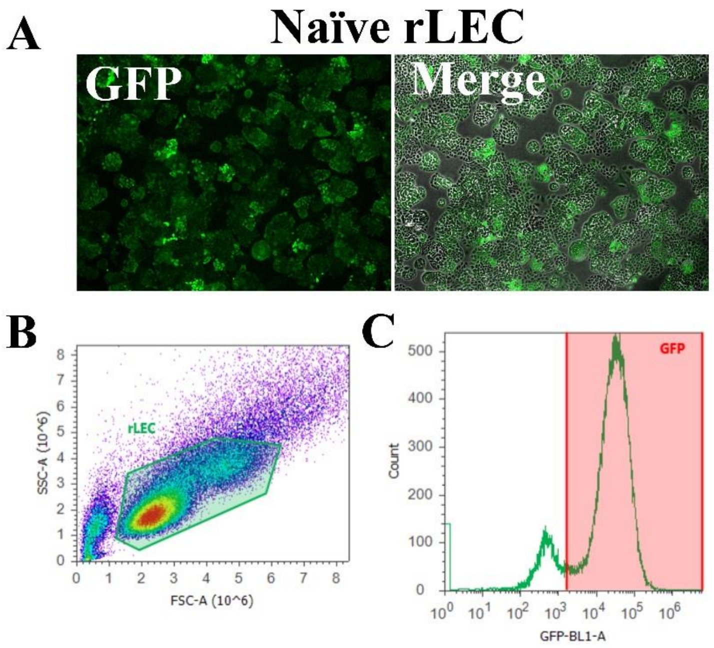

Figure S2. Transduction of naïve rLEC with blank GFP control lentiviral vector. A) Transduced naïve rLEC expressing only GFP. Merge image includes corresponding phase contrast microscopy. Scale bar: 500 μM. B-C) Flow cytometric analysis of puromycine-purified rLEC after lentiviral transduction with GFP control vector shows more than 80% GFP-positivity.

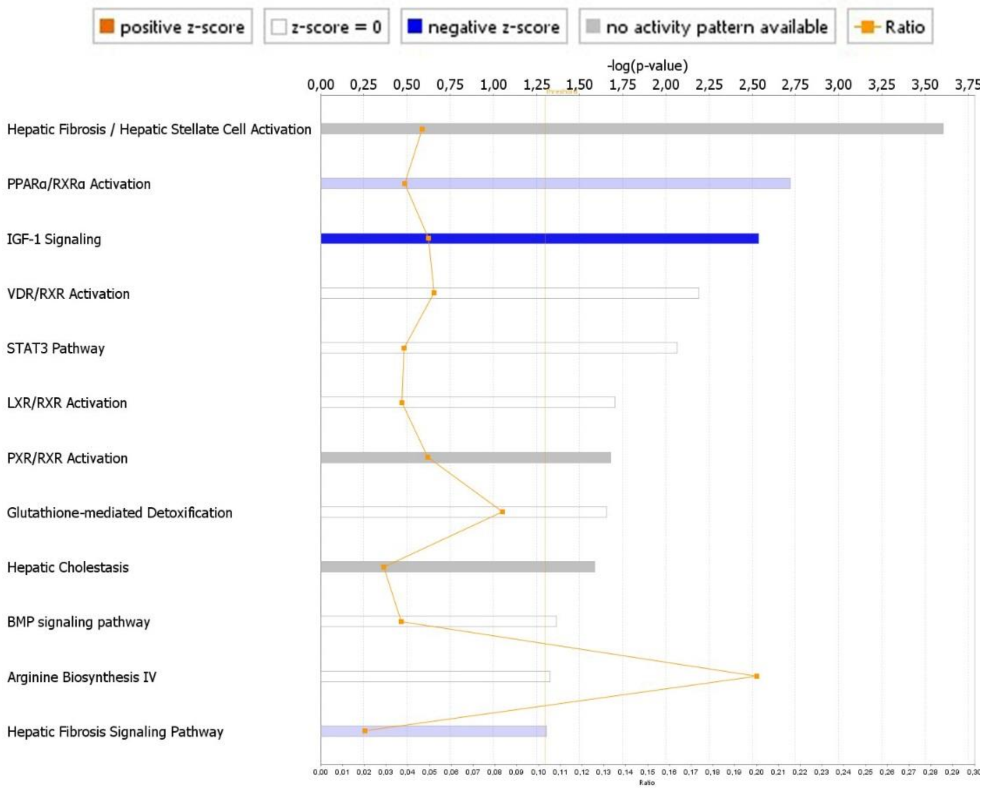

Figure S3. Canonical pathways modulated in HNF4α-transduced rLEC. Ingenuity Pathway Analysis Software was used to identify to which canonical pathways the altered genes by HNF4α in naïve rLEC belong. The '*IGF-1 Signaling*' pathway was predicted to be negatively influenced (z-score < -2; p-value < 0.05) by HNF4α over-expression in naïve rLEC.
